# A simple method to make a reusable volar splint

**DOI:** 10.4103/0970-0358.44931

**Published:** 2008

**Authors:** Roba Khundkar, Emily West

**Affiliations:** Department of Plastic Surgery, Frenchay Hospital, Frenchay, Bristol, BS8 1SY UK

Sir,

Application of a Plaster of Paris (POP) volar splint for the immobilization and/or patient comfort is a common procedure in Plastic Surgery Units either after the initial assessment of a trauma patient or postoperatively in theater. In our unit, the on-call plastic surgery trainee may assess up to 30 new referrals in a day. Most of these are hand trauma referrals, often requiring a volar resting slab to be applied by the trainee. However, patients often need senior review/reassessment within 24 h, necessitating the removal and reapplication of this cast.

The standard plaster technique requires wrapping wool around the limb, placing the wet plaster on this, and securing it with a circumferential crepe bandage. When removed, this is not easily reusable.

We describe here a simple method to make a reusable cast using standard plaster trolley materials. This method utilizes a stockinette to confine the POP, preventing it from sticking to the wool.

**Figure 1:**
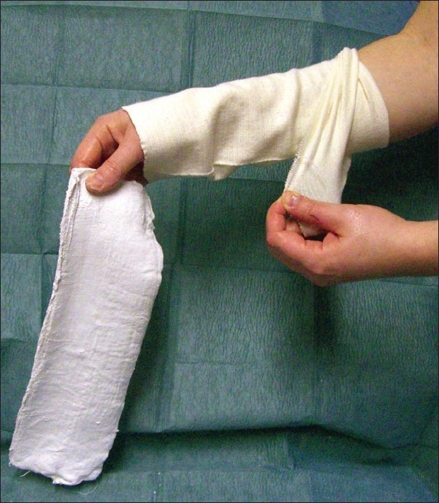
Stockinette being pulled over plaster

**Figure 2:**
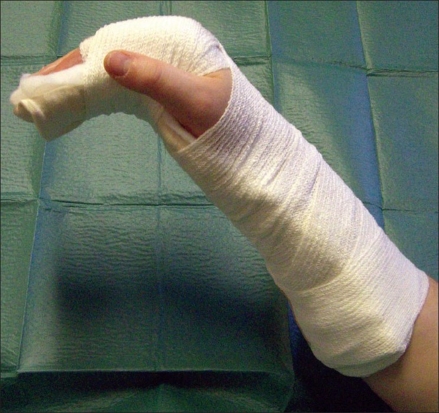
Plaster secured to arm with bandage and tape

- Cut the required length of plaster as usual.
- Cut a length of stockinette approximately 2 inches longer than the plaster. Pull this piece of stockinette over your own dominant forearm.
- The plaster is soaked in warm water and wrung out. While holding the plaster with your dominant hand, pull the stockinette over the plaster [Figure 1].
- Even out the plaster inside the stockinette by laying it on a flat surface.
- Fold the edges of the stockinette inwards to neaten the ends.
- Lay wool padding over the top and apply to the volar aspect as required. Secure to arm with bandage and tape [Figure 2].
- Mould into required position as it sets.

The standard POP cast is not easy to reuse. The method described above allows the patient to have a removable cast that can be reapplied after reassessment simply by using a new piece of wool and crepe.

- Saves time during on-call periods.
- Can be reused postoperatively, saving time in the theater
- Cost-saving, with single rather than multiple POPs required.
- More aesthetically pleasing as the POP is confined within the stockinette, giving it a neater appearance

